# Detection of Pine Wilt Disease in UAV Remote Sensing Images Based on SLMW-Net

**DOI:** 10.3390/plants14162490

**Published:** 2025-08-11

**Authors:** Xiaoli Yuan, Guoxiong Zhou, Yongming Yan, Xuewu Yan

**Affiliations:** 1 Institute of Artificial Intelligence Application, Central South University of Forestry and Technology, Changsha 410004, China; yyuanxli@163.com (X.Y.); zhougx01@163.com (G.Z.); 2 Hunan Academy of Forestry, Changsha 410018, China

**Keywords:** pine wilt disease, SLMW-Net, SFEM, MFAM, WLIoU Loss

## Abstract

The pine wood nematode is responsible for pine wilt disease, which poses a significant threat to forest ecosystems worldwide. If not quickly detected and removed, the disease spreads rapidly. Advancements in UAV and image detection technologies are crucial for disease monitoring, enabling efficient and automated identification of pine wilt disease. However, challenges persist in the detection of pine wilt disease, including complex UAV imagery backgrounds, difficulty extracting subtle features, and prediction frame bias. In this study, we develop a specialized UAV remote sensing pine forest ARen dataset and introduce a novel pine wilt disease detection model, SLMW-Net. Firstly, the Self-Learning Feature Extraction Module (SFEM) is proposed, combining a convolutional operation and a learnable normalization layer, which effectively solves the problem of difficult feature extraction from pine trees in complex backgrounds and reduces the interference of irrelevant regions. Secondly, the MicroFeature Attention Mechanism (MFAM) is designed to enhance the capture of tiny features of pine trees infected by initial nematode diseases by combining Grouped Attention and Gated Feed-Forward. Then, Weighted and Linearly Scaled IoU Loss (WLIoU Loss) is introduced, which combines weight adjustment and linear stretch truncation to improve the learning strategy, enhance the model performance and generalization ability. SLMW-Net is trained on the self-built ARen dataset and compared with seven existing methods. The experimental results show that SLMW-Net outperforms all other methods, achieving an mAP@0.5 of 86.7% and an mAP@0.5:0.95 of 40.1%. Compared to the backbone model, the mAP@0.5 increased from 83.9% to 86.7%. Therefore, the proposed SLMW-Net has demonstrated strong capabilities to address three major challenges related to pine wilt disease detection, helping to protect forest health and maintain ecological balance.

## 1. Introduction

Pine forests are an extremely important component of forest ecosystems [1], playing a vital role in promoting biodiversity, soil and water conservation, and climate regulation [2]. Additionally, pine trees provide various resources such as timber and resin, possessing significant economic value and being widely utilized in industries such as construction, papermaking, and chemicals [3]. However, pine wilt disease is commonly referred to as cancer in pine forests. It is caused by a pathogen pine wood nematode and is capable of killing trees quickly and rapidly [4]. Vectors such as the infected Serragon genus of insects, like Monochamus alternatus, are responsible for transmitting the disease. Pine wilt disease is highly contagious [5]. If a pine tree is infected by a nematode, it quickly spreads throughout in the trunk of the pine tree and kills it by destroying the movement of water and nutrients. Pine wilt disease is spreading at explosive speed [6], and tends to devastate pine forests within three to five years [7]. Pine wilt disease represents a critical threat to the sustainable management of pine forests worldwide, posing particularly significant concerns across regions such as Asia, Europe, and North America [8]. Given its high mortality rate and strong transmissibility, early detection and diagnosis are essential. Only with accurate and efficient detection technologies can timely control measures be implemented, thereby reducing the ecological and economic impact of the disease [9].

Traditional detection of pine wilt disease has primarily relied on manual field surveys conducted by forestry professionals through on-site inspections [10]. Although such approaches provide direct and intuitive observations, they are constrained by several inherent limitations, including high labor intensity, sensitivity to complex terrain, and low operational efficiency. These drawbacks severely restrict their scalability for large-scale forest monitoring and hinder timely and accurate diagnosis [11]. To overcome these limitations, advancements in machine learning have spurred increasing interest in integrating UAV remote sensing imagery with classical machine learning algorithms, aiming to enable automated detection and assessment of pine wilt disease caused by the pine wood nematode [12,13,14,15]. While these approaches have enhanced automation to a certain extent, they remain dependent on manually engineered features and the selection of appropriate classifiers, limiting their adaptability to heterogeneous and dynamic forest environments and thus constraining their practical application [16]. In recent years, the rapid evolution of deep learning techniques has led to their growing application in pine wilt disease detection. Compared with traditional methodologies, deep learning models possess the capability to automatically extract high-level features from large-scale raw datasets, rendering them more robust in handling complex backgrounds and diverse forest scenarios [17]. This capacity offers substantial technical support for the intelligent and automated detection of forest diseases, greatly promoting their implementation in real-world forest management practices.

At present, object detection approaches based on deep learning are generally divided into two main categories: two-stage and one-stage models. Two-stage models, represented by the R-CNN series [18], usually begin by generating candidate regions, followed by separate classification and bounding box regression. These models offer high detection accuracy but are limited by inference speed and computational efficiency. In contrast, one-stage detection models, such as the SSD series proposed by Liu, W et al. [19] and the YOLO series by Redmon, J et al. [20] are more widely applicable in practical applications due to their advantages of simple structure and fast speed, and are particularly suitable for tasks with high real-time requirements. As a representative one-stage detection framework, the YOLO series has undergone continuous optimization across versions and has become a mainstream general-purpose object detection tool. As early as 2017, YOLOv2 [21] attracted widespread attention for its superior feature extraction capabilities and high detection efficiency. Subsequently, Wu, K et al. [22] improved YOLOv3 for pine wilt disease detection by introducing the Darknet backbone, a logical classifier, and the BCE loss function, significantly enhancing the accuracy of infected tree identification. Building on this, YOLOv4 [23] further optimized its architecture by incorporating a BoF and BoS, including data augmentation techniques such as CutMix, CutOut, and Mixup. These enhancements improved detection accuracy without significantly increasing inference cost. The model also integrated non-linear activation functions and skip connections, boosting its feature representation capabilities. Based on this framework, Sun, Z et al. [24] expanded the backbone network by integrating the Inceptionv2 structure, achieving improvements in both detection efficiency and accuracy while reducing the number of parameters. With continued research, Wang, L et al. [25] integrated the SE-Net into the YOLOv5 framework, significantly enhancing its feature extraction capability. Additionally, they introduced a BiFPN to improve perception and adaptability to multi-scale objects in complex scenes. Building on this, Ye, X et al. [26] combined YOLOv5 with the StrongSORT algorithm to realize dynamic visual tracking and counting of infected pine trees, expanding the model’s practical application in intelligent forestry. The subsequent YOLOv7 [27,28] introduced the E-ELAN, which significantly improved feature learning efficiency and training performance while maintaining model lightweight characteristics. Following this, YOLOv8 [29] further innovated by incorporating anchor-free detection mechanisms, the C2f module, an improved decoupled head structure, and a more efficient loss function. These improvements led to a notable increase in detection accuracy while preserving the model’s strength in real-time inference performance. As a result, research on optimizing YOLOv8 continues to emerge, aiming to further improve its performance for specific tasks. For example, Xu, S et al. [30] enhanced the convolutional network’s expressive power by introducing ODConv, designed a DyHead module for precise PWD feature extraction, and adopted the MPDioU regression mechanism to improve the accuracy and efficiency of bounding box prediction. Additionally, Yao, J et al. [31] developed the Pine-YOLO model based on YOLOv8, integrating DSConv and MCA, which significantly improved the robustness in complex forestry scenarios. Building on this series of advancements, YOLOv11 [32] achieved further breakthroughs in architecture design by incorporating the C3k2 module, the SPPF structure, and the C2PSA component. These enhancements substantially improved feature extraction capabilities and overall performance, providing stronger technical support for object detection tasks.

Overall, despite significant progress in detecting pine wilt disease, several challenges remain. First, remote sensing images captured by UAV often have complex backgrounds: Changes in lighting, the presence of image noise, and visual similarities between unrelated objects such as buildings, water bodies, and affected trees pose challenges to accurately identifying pine trees in the scene. These factors can increase the possibility of detection errors and hinder the overall effectiveness of the model. Second, it remains difficult to extract the subtle features of infected pine trees. The early symptoms of pine wilt disease are often mild, and the pathological changes on the bark and branches are hard to recognize. Due to the lack of effective attention mechanisms, existing models struggle to accurately capture and identify these subtle disease features. Third, prediction box deviations are common. When infected pine trees are densely distributed, conventional detection algorithms often generate a large number of redundant prediction boxes, and the loss functions fail to account for directional mismatches between predicted and ground-truth boxes. This may lead to inaccurate localization of infected trees, thereby reducing training efficiency and diminishing detection accuracy.

In light of these challenges, our primary research objective is to address three key issues in pine wilt disease detection: severe background interference, difficulty in extracting subtle features, and deviations in predicted bounding boxes. To achieve this objective, we selected the YOLOv11 model, which is known for its strong accuracy and adaptability, as the baseline network. Building upon this foundation, we developed a new detection model called SLMW-Net for identifying pine wilt disease. The main contributions of this study are summarized as follows:

(a) A high-resolution ARen dataset is developed to include trees infected by pine wood nematodes. The dataset consists of 750 annotated UAV images for training and testing purposes. This dataset captures a wide range of forest conditions, such as bare soil, red broadleaf species, dead trees, and various background disturbances, ensuring a comprehensive representation of complex environmental factors.
(b) A Self-Learning Feature Extraction Module (SFEM) is proposed that contains a convolution block and a learnable normalization layer. This design improves the discriminatory representation of pine wilt disease features through normalization while effectively retaining the original local details of the input. It is very efficient at extracting characteristics of nematode-infected pine trees, even in scenarios with complex ground conditions and significant vegetation interference.
(c) A MicroFeature Attention Mechanism (MFAM) is introduced, combining Grouped Attention with a Gated Feed-Forward network. This method significantly improves the ability to capture pine wood nematode characteristics and also enhances the overall accuracy of feature representation. By overcoming the limitations of traditional attention mechanisms in detecting microscopic disease characteristics, this method greatly improves the accuracy of pine wood nematode detection.
(d) A Weighted and Linearly Scaled IoU Loss (WLIoU Loss) is designed to further enhance the training process. It is specifically tailored for pine trees infected with pine wilt disease. By regulating multifaceted factors, the WLIoU Loss function surpasses category imbalance and hard sample detection challenges and enhances positive sample weighting by stretching and truncation mechanisms, thereby effectively addressing the issue of biased prediction boxes.

## 2. Datasets and Methods

### 2.1. Data Acquisition

**Figure 1:**
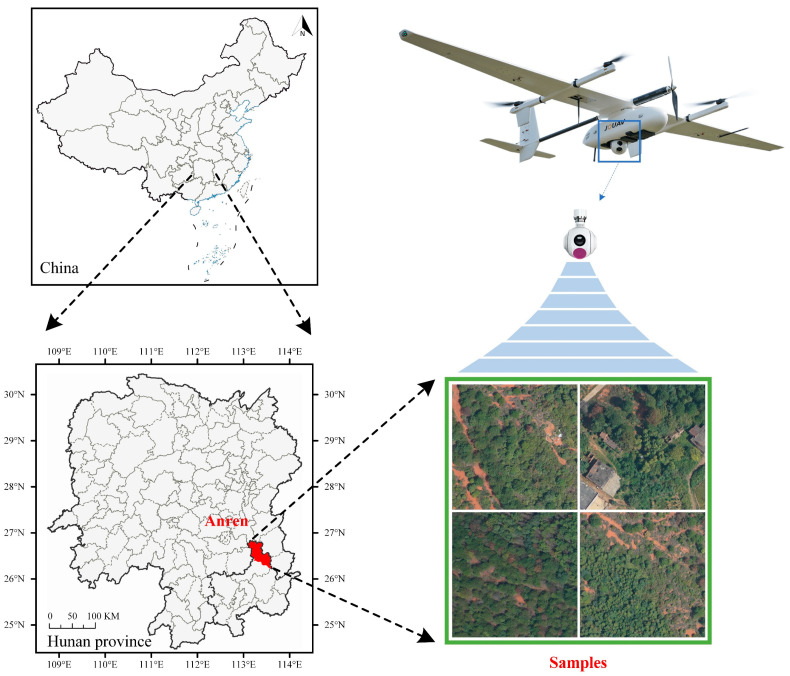
Data acquisition.

The ARen dataset for pine wilt disease used in this study was obtained from the Hunan Academy of Forestry, China. The dataset collection area is located in Anren County, Chenzhou City, Hunan Province, with geographic coordinates ranging from 113°05′ to 113°36′ east longitude and 26°17′ to 26°51′ north latitude. This region, located in the southeast of Hunan Province, has a typical subtropical monsoon climate. The main forest types include slow-growing broadleaf forests, horsetail pine, wetland pine, and others, with pine forests being the predominant type. The infection severity of pine trees is notably high, making this area a typical representative region for pine wilt disease. The pine trees within the study area differ in age and are unevenly distributed both vertically and horizontally. In terms of vertical structure, the forest is stratified into three distinct layers: the top layer features tall, mature pines forming the canopy; the middle layer includes moderately sized pines along with various other tree species; and the bottom layer is primarily occupied by shrubs and herbaceous vegetation. In terms of horizontal structure, the trees in the forest are unevenly distributed, with alternating dense clusters and open spaces, highlighting the complexity and diversity of the forest structure. Data collection took place between September and October 2024. During this period, most pine trees infected with pine wilt disease exhibited typical reddish-brown symptoms, while other broadleaf trees showed no significant changes. As such, this period provided the best opportunity for capturing the most visible symptoms of pine wilt disease. We used the CW-15 UAV as the flight platform, with a flight altitude of approximately 500 m and a ground sampling distance (GSD) of 10 cm. It was equipped with a full-frame 61-megapixel sensor and a 35 mm focal length lens. Figure 1 illustrates the data acquisition.

### 2.2. Data Processing

Due to the wide coverage of the UAV-captured imagery, each original image was cropped to 800 × 800 pixel tiles. Under the professional guidance of forestry experts, 750 image patches were selected after filtering, retaining those containing areas of affected pine trees and excluding images in which vegetation features could not be accurately identified due to factors such as cloud cover, strong shadows, or image blurring. These images were then randomly divided into training and validation sets in a ratio of 8:2, with the training set containing 600 images and the validation set containing 150 images. For image annotation, forestry experts manually labeled the data using the LabelImg software 1.8.6. Specifically, images of pine wilt disease were imported into the LabelImg platform, where experienced foresters delineated diseased regions through manual annotation. The resulting labels were subsequently saved in txt format.

### 2.3. SLMW-Net

**Figure 2:**
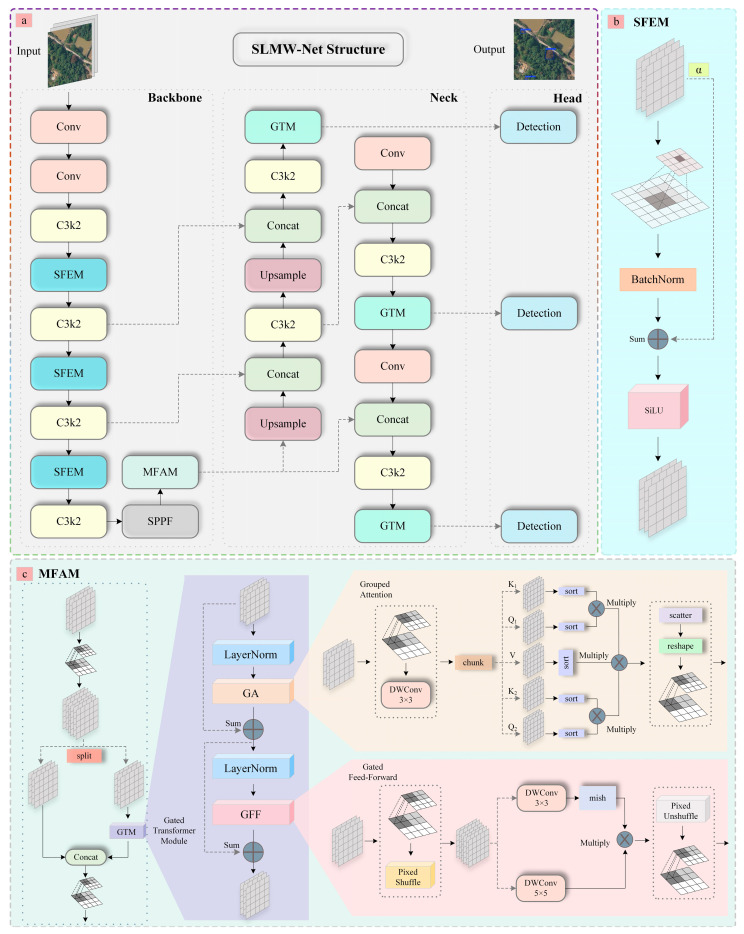
Structure of SLMW-Net. (**a**) The backbone, neck, and head of SLMW-Net; (**b**) shows the structure of SFEM; (**c**) shows the structure of MFAM.

In this research, we introduce SLMW-Net, a target detection method based on YOLOv11, designed to efficiently and accurately detect pine wilt disease in natural settings. The model integrates the SFEM and MFAM modules and employs the WLIoU Loss to compute model loss, guiding the optimization of model parameters and thereby enhancing training effectiveness. These improvements collectively boost the efficiency of pine wilt disease detection, ensuring accurate recognition even under challenging conditions such as complex backgrounds or subtle disease features. As depicted in Figure 2, its architecture is composed of three principal parts: the backbone, the neck, and multiple head detection modules. Inside the backbone, we add the SFEM block after three C3k2 blocks to enhance feature extraction, aiding in the capture of characteristic information specific to pine wilt disease and addressing the challenges posed by complex backgrounds. Following processing by the SPPF block, the feature map is passed into the MFAM module to further refine the representation of input features. By applying Grouped Attention, Gated Feed-Forward, and depthwise separated convolution, the model can capture subtle disease features more effectively, focusing on regions with minor disease manifestations, which significantly improves detection accuracy. In the neck, prior to the output of each detection head, we incorporate the Gated Transformer Module to further enhance feature fusion, enabling the network to learn increasingly complex representations of disease features.

#### 2.3.1. Self-Learning Feature Extraction Module (SFEM)

In the detection of pine wilt disease, the convolution operation serves as the core computational module in neural networks. It selects the most significant local features by filtering within the receptive field [33], which are critical for disease identification [34]. However, in UAV remote sensing images, the complex and variable background makes it challenging for traditional single-scale convolutions to capture features across different spatial scales, resulting in incomplete information extraction. Additionally, commonly used normalization techniques often lack sufficient adaptability to image noise and illumination variations, further limiting model performance. To address it, we introduce a self-learning feature extraction module (SFEM), designed to enhance feature representation and improve the efficiency of subsequent deployment. SFEM comprises a convolutional layer and a learnable normalization layer, which together progressively strengthen the ability to extract pine wilt disease-related features across multi-layer feature maps. This approach not only preserves the original local details of the inputs but also reconstructs a more discriminative feature representation through normalization. The design is particularly effective in environments with complex ground conditions and significant vegetation disturbance.

To capture feature information from the input image, we apply SFEM prior to the feature output at each scale within the backbone of YOLOv11. The SFEM structure is depicted in Figure 2b, and its specific processing flow is as follows. The feature extraction of the base convolution is performed first, where X denotes the input feature map, ∗ denotes the 2D convolution, W denotes the convolution kernel parameter, and Y denotes the convolution output.

(1) $$Y=Conv\left(X\right)=W*X$$

Subsequently, a learnable reparameterization batch normalization LeaBN is introduced in the normalization process, where $\mu_{c}$ and $\sigma_{c}^{2}$ denote the mean and variance of each channel. $\gamma_{c}$ and $\beta_{c}$ denote the scaling and translation parameters of each channel. α is a self-learning parameter, initially set to 1, and is optimized during training to control the proportion of direct inputs and retain feature information. Specifically, α will be added to the model’s list of trainable parameters, incorporated into the computation graph during forward propagation, participate in backpropagation, and subsequently be updated automatically based on the gradients.

(2) $$LeaBN\left(Y_{C,H,W}\right)=\gamma_{c}\times \frac{Y_{C,H,W}-\mu_{c}}{\sqrt{\sigma_{c}^{2}+\varepsilon }}+\beta_{c}+\alpha \times Y_{C,H,W}$$

(3) $$Z=LeaBN\left(Y_{C,H,W}\right)$$

Finally, activation is performed using the SiLU function to obtain nonlinear relationships of the input features, so that our model can obtain more details about the features.

(4) $$Output=SiLU\left(Z\right)=Z\times \sigma \left(Z\right)$$

In conclusion, this module enhances the local features of the diseased spots during feature extraction, effectively suppresses the random noise in the background, improves the ability to focus on the pine wood nematode-infested area, and improves the model performance.

#### 2.3.2. MicroFeature Attention Mechanism (MFAM)

Some infected pine trees initially exhibit subtle signs of nematode infection, which are often challenging for traditional models to detect due to the difficulty in extracting these minute features. Consequently, accurately identifying and extracting these subtle traits is essential for effectively detecting pine wilt disease. Drawing inspiration from human visual processing mechanisms, attention mechanisms enable neural networks to more accurately extract key features by assigning weighted coefficients to them [35]. Attention mechanisms are now commonly integrated into convolutional neural networks (CNNs) [36], graph convolutional networks (GCNs) [37], and other neural network architectures. As attention mechanisms continue to develop, traditional methods have shown significant limitations. These methods often focus on wider areas and tend to ignore key details, especially when identifying subtle signs of pine wilt disease. To solve these problems, we introduced the MicroFeature Attention Mechanism (MFAM). This module has significant advantages in identifying complex and diverse features and can accurately detect weak symptoms of pine wilt disease while maintaining high adaptability and robustness.

Figure 2c depicts the architecture of MFAM. Clearly visible is that MFAM employs a number of important such as the Gated Transformer Module (GTM), Grouped Attention (GA), Gated Feed-Forward (GFF), and Depthwise Separable Convolution (DWConv). By leveraging the collaborative operation of these modules, MFAM effectively responded to the challenge of detecting subtle features in the early stages of pine wilt disease. It significantly improves the characterization of these minor disease features in UAV remote sensing images, allowing the model to capture disease-related information more accurately and in detail. Specifically, the GTM combines the benefits of Grouped Attention and the Gated Feed-Forward network. The GA module strengthens attention to subtle features by dynamically adjusting attention weights, thereby highlighting key disease indicators and filtering out irrelevant information. The GFF module helps to better capture the spatial details of pine wilt disease characteristics, gradually extracts edge, texture, and shape information through hidden channels, while abstracting more complex features at a deeper level. This contributes to enhanced detection accuracy and robustness, particularly for early stage disease features. Furthermore, DWConv reduces information loss and computational complexity while enhancing feature representation, allowing the network to efficiently process large-scale remote sensing images. The mish activation function mitigates issues of gradient vanishing and dead neurons, bolstering feature learning capability. Its smooth gradient and nonlinear transformation enhance the ability to capture early symptoms of pine wilt disease. The processing flow of MFAM is outlined below.

The MFAM uses a staged partial structure that allows only a portion of the channels to pass through the complex GTM, which reduces the amount of computation while retaining a portion of the original feature channels, which helps information fusion and the flow of the gradient. In the MFAM, the input is $X\in {\mathbb{R}}^{B\times C\times H\times W}$, the input is first processed with ascending convolution, followed by dimensional splitting, and then one part is left unprocessed, and the other part goes into the GTM after processing. The two parts are then spliced, and finally, dimensionality reduction is performed to return to the original feature map size. Where DX is the result of dimension upgrading, A and B are the two parts after splitting according to the channel, with the same dimension of X. The results of splicing and downscaling is CX.

(5) $$DX=Conv1\times 1_{in\to 2C}\left(X\right)$$

(6) DX=[A;B]

(7) $$B^{\prime }=GTM\left(B\right)$$

(8) $$CX=Conv1\times 1_{2C\to C}\left(\left[A;B^{\prime }\right]\right)$$

The Gated Transformer Module (GTM) consists of Grouped Attention (GA) and Gated Feed-Forward (GFF). The processing flow is outlined below. For the input feature B generates Q, K, V, where DWConv represents depthwise separated convolution and chunk divides the channels into five parts, each with the channel number of C.

(9) $$QKV_{raw}=DWConv_{3\times 3}\left(Conv1\times 1_{C\to 5C}\left(B\right)\right)\in {\mathbb{R}}^{B\times 5C\times H\times W}$$

(10) $$\left(Q_{1},K_{1},Q_{2},K_{2},V\right)=chunk\left(QKV_{raw},5;\mathrm{along}\;\mathrm{channel}\right)$$

Sorting is then performed by first flattening V to ${\mathbb{R}}^{B\times C\times (H\times W)}$, in ascending order. Then, $Q_{1}$, $K_{1}$, $Q_{2}$, and $K_{2}$ are reordered by indexing operations to ensure that they are consistent with V.

(11) $$\left(V_{sorted},idx\right)=sort\left(V,\dim=2\right)$$

The attention calculation is tuned by the temperature parameter, which controls the sensitivity of the similarity between the features, performs dynamic range adjustment, and calculates the result O by multiplying with $V_{sorted}$. The role of the temperature parameter is to control the degree of smoothing of the scores. A higher temperature results in a smoother attention distribution, while a lower temperature leads to a sharper attention distribution. $d_{k}$ indicates the size of the key vector.

(12) $$Attention=\frac{Q\times K^{T}}{\sqrt{d_{K}}}\times temperature$$

(13) $$O=Attention\times V_{sorted}$$

Subsequently, a scatter operation is performed based on the previously obtained idx to restore them to the original pixel positions, and finally, the reshape is returned to ${\mathbb{R}}^{B\times C\times H\times W}$.

(14) $$Output=Conv1\times 1_{C\to C}\left(\mathrm{Reshape}\left(O\right)\right)$$

In Gated Feed-Forward (GFF), for the input $X\in {\mathbb{R}}^{B\times C\times H\times W}$, the dimensionality convolution is performed first. Where the channel dimension expansion factor is r, the number of hidden channels $C_{h}=r\times C$.

(15) $$U=Conv1\times 1_{C\to 2C_{h}}(X)\in {\mathbb{R}}^{B\times 2C_{h}\times H\times W}$$

Then, PixelShuffle upsampling is performed, a step that moves the spatial information in the channel dimensions onto H and W, with the spatial resolution scaled up by a factor of two and channel’s number scaled down by a factor of four. And the channel is split to form two branches, $U_{1}^{\prime }$ and $U_{2}^{\prime }$. Large kernel convolution is performed for branch $U_{1}^{\prime }$ and dilation convolution is performed for branch $U_{2}^{\prime }$.

(16) $$U^{\prime }=PixedShuffle(U)\in {\mathbb{R}}^{B\times \frac{C_{h}}{2}\times (2H)\times (2W)}$$

(17) $$U^{\prime }=[U_{1}^{\prime };U_{2}^{\prime }]$$

(18) $$\left\{\begin{array}{l} V_{1}=DWConv_{5\times 5}\left(U_{1}^{\prime }\right)\\ V_{2}=DWConv_{3\times 3,d=2}\left(U_{2}^{\prime }\right) \end{array}\right.$$

Then, a nonlinear activation of the second branch is conducted and then multiplied element by element with the first branch to complete the two-scale gated fusion, where mish is involved as a smooth nonmonotonic activation function.

(19) $$\left\{\begin{array}{l} mish\left(V_{2}\right)=V_{2}\tanh\left(\ln\left(1+e^{V_{2}}\right)\right)\\ G=mish\left(V_{2}\right)\times V_{1} \end{array}\right.$$

By downsampling with PixelUnshuffle, the spatial information is moved back to the channel dimension, the spatial resolution is restored, and the channel’s number is scaled up by a factor of four. Finally, using convolution, we project the channel’s number back to the input size C.

(20) $$G^{\prime }=PixedUnShuffle\left(G\right)\in {\mathbb{R}}^{B\times C_{h}\times H\times W}$$

(21) $$Output=Conv1\times 1_{C_{h}\to C}\left(G^{\prime }\right)\in {\mathbb{R}}^{B\times C\times H\times W}$$

The MFAM module enables the model to accurately identify lesion areas associated with pine wilt disease by modeling multi-level image features. Even if the differences in these areas are subtle, the model can efficiently capture this information through Grouped Attention and Gated Feed-Forward mechanisms. This method effectively solves the problem of early identification of subtle features of pine wilt disease, and provides strong characterization and learning capabilities for detecting subtle disease features in UAV remote sensing images.

#### 2.3.3. Weighted and Linearly Scaled IoU Loss (WLIoU Loss)

The loss function is crucial in deep learning to measure the difference between actual labels and model predictions, and it directly affects the process of updating and optimizing network parameters [38]. In image detection tasks, selecting the appropriate loss function is critical because it will directly affect the model’s fitting effect on training data and play a key role in optimization goals [39]. Therefore, the loss function occupies a core position in neural network model training. For target detection tasks related to pine wilt disease, we chose the Intersection over Union (IoU) loss function because it can effectively handle difficult samples [40]. Building on this, we propose a mixed loss function, the Weighted and Linearly Scaled IoU Loss (WLIoU Loss), which combines IoU sample weight adjustment and linear stretch truncation. Compared to traditional loss functions, WLIoU significantly enhances model learning efficiency by modifying the weights between positive and negative samples. By optimizing the weighting parameters, this method effectively balances fitting ability and generalization, while mitigating the risk of overfitting. The specific processing flow of the WLIoU loss function is outlined below.

IoU calculates the overlap between the predicted bounding box and the ground truth box. In this case, inter represents the intersection area, while union refers to the union area.

(22) $$IoU=\frac{\mathrm{inter}}{\mathrm{union}}$$

The minimum outer box diagonal length calculation is performed first. $C_{w}$ and $C_{h}$ denote the size of the minimum outer rectangle that encloses both the real box and the predicted box. Where $\left(x_{p-ul},y_{p-ul}\right)$ and $\left(x_{p-lr},y_{p-lr}\right)$ are the upper-left and lower-right coordinates of the prediction frame, and $\left(x_{r-ul},y_{r-ul}\right)$ and $\left(x_{r-lr},y_{r-lr}\right)$ are the upper-left and lower-right coordinates of the real frame.

(23) $$\left\{\begin{array}{l} C_{w}=\max\left(x_{p-lr},x_{r-lr}\right)-\min\left(x_{p-ul},x_{r-ul}\right)\\ C_{h}=\max\left(y_{p-lr},y_{r-lr}\right)-\min\left(y_{p-ul},y_{r-ul}\right)\\ C^{2}=C_{w}^{2}+C_{h}^{2} \end{array}\right.$$

Next, the squared Euclidean distance is computed between the centers of the predicted frame and the real frame, where Δx and Δy are the differences between the coordinates of the two frame centers.

(24) $$\left\{\begin{array}{l} \Delta x=\frac{\left(x_{r-ul}+x_{r-lr}\right)-\left(x_{p-ul}+x_{p-lr}\right)}{2}\\ \Delta y=\frac{\left(y_{r-ul}+y_{r-lr}\right)-\left(y_{p-ul}+y_{p-lr}\right)}{2}\\ \rho^{2}=\Delta x^{2}+\Delta y^{2} \end{array}\right.$$

The Aspect Ratio Penalty Term is used to measure the difference in aspect ratios between two boxes. Here, the predicted box’s width and height is represented by $\left(w_{1},h_{1}\right)$, while the ground truth box’s width and height is represented by $\left(w_{2},h_{2}\right)$. The normalization factor $\frac{4}{\pi^{2}}$ ensures that $v\in \left[0,1\right]$, and the coefficient $\alpha_{1}$ dynamically adjusts the weight of the aspect ratio difference in the overall loss.

(25) $$v=\frac{4}{\pi^{2}}{\left(\arctan\frac{w_{2}}{h_{2}}-\arctan\frac{w_{1}}{h_{1}}\right)}^{2}$$

(26) $$\alpha_{1}=\frac{v}{\left(1-IoU\right)+v}$$

$IoU^{\prime }$ is calculated by subtracting the center distance penalty and the aspect ratio penalty. Subsequently, the moderating factor κ is increased, decreasing its loss weight for easy-to-detect samples (high IoU) and increasing attention for difficult samples (low IoU).

(27) $$IoU^{\prime }=IoU-\left(\frac{\rho^{2}}{c^{2}}+v\times \alpha_{1}\right)$$

(28) $$w_{f}={\left(IoU\right)}^{\kappa }$$

(29) $$IoU^{\prime \prime }=\left(1-IoU^{\prime }\right)\times w_{f}$$

Finally, a linear stretching and truncation is performed to amplify the gradient signal in the high IoU interval and compress the very small value interval. Where d=0.00, u=0.95, and $\sum_{i}w_{i}$ denotes the normalization to the sum of all positive sample weights.

(30) $$suIoU=clamp\left(\frac{IoU^{\prime \prime }-d}{u-d}\right)$$

(31) $$L_{WLIoU}=\frac{1}{\sum_{i}w_{i}}\sum_{i}w_{i}\left(1-suIoU\right)$$

WLIoU Loss takes into account overlap, position, and shape, as well as the focusing and weighting of difficult samples, which is more beneficial for addressing class imbalance and difficult samples, while simultaneously optimizing multiple objectives.

## 3. Results

### 3.1. Experimental Environment and Training Details

In this study, all experiments were conducted in the same hardware and software environments to avoid different experimental conditions affecting the results of SLMW-Net. AMD EPYC 9754 128-Core Processor CPU and NVIDIA GeForce RTX 4090 D GPU were the main hardware used in this experiment. Table 1 shows the experimental environment parameters. The experiment was set up with 300 training epochs, using SGD as the optimizer, an initial learning rate of 0.01, and a learning rate decay factor of 0.01. Other hyperparameters were set as follows: batch size 4, momentum value 0.937, and weight decay coefficient 0.0005. During the training phase, we employed WLIoU Loss as the loss function, updating parameters by calculating the model loss to accelerate convergence and enhance stability.

### 3.2. Evaluation Indicators

This study use indicators such as mAP@0.5, precision, recall, and mAP@0.5:0.95 to evaluate the effectiveness of the model. The relevant equations are given below.

As a comprehensive indicator, Mean Average Precision (mAP) is calculated by averaging individual Average Precision (AP) scores. The AP indicator measures the area under the precision-recall curve, which describes the balance between precision and recall at different thresholds, so mAP can provide a more comprehensive assessment of the performance of the detection model. Specifically, mAP@0.5 indicates the mean average precision with an IoU threshold of 0.5, whereas mAP@0.5:0.95 denotes the average mAP calculated in steps of 0.05 at IoU thresholds from 0.5 to 0.95.

(32) $$mAP=\frac{\sum_{N}^{i=1}AP_{i}}{N}$$

Precision reflects the likelihood of accurately identifying the target. TP refers to the correct detection of a diseased tree, while FP signifies the misdetection of a healthy pine tree as diseased.

(33) $$Precision=\frac{TP}{TP+FP}$$

Recall represents the likelihood of correctly identifying a diseased pine tree. FN refers to the incorrect prediction of a diseased tree as healthy.

(34) $$Recall=\frac{TP}{TP+FN}$$

### 3.3. Model Performance Analysis

**Figure 3:**
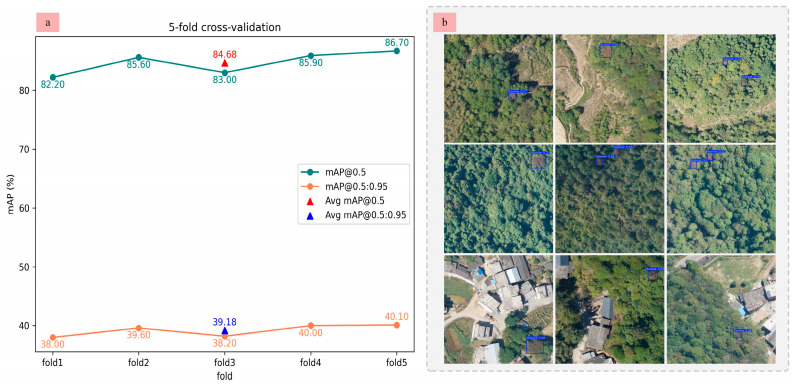
Cross-validation. (**a**) 5-fold cross-validation on ARen dataset; (**b**) field-based cross-validation on Jiuyi Mountain.

To comprehensively evaluate the spatiotemporal generalization capability of the proposed model in practical applications, we first conducted a 5-fold sample-based cross-validation. The entire dataset was uniformly divided into five equal subsets. In each iteration, one subset was used for validation while the remaining four were used for training. This process was repeated five times to ensure a balanced and robust assessment. For each fold, the model’s best performance was retained, and the final detection accuracy was reported as the average across all folds. As shown in Figure 3a, SLMW-Net demonstrates stable and consistent performance across different data partitions, indicating strong generalization under random sampling conditions. In addition, to further evaluate the model’s spatial transferability, we conducted an independent field-based cross-validation. Specifically, we trained the model using the ARen dataset and tested it on UAV imagery collected from Jiuyi Mountain in Yongzhou City, Hunan Province, which features distinct forest and terrain conditions. The results, presented in Figure 3b, show that SLMW-Net maintains satisfactory detection accuracy and robustness when applied to this geographically distinct region, further validating its potential for real-world deployment in diverse pine forest environments.

**Figure 4:**
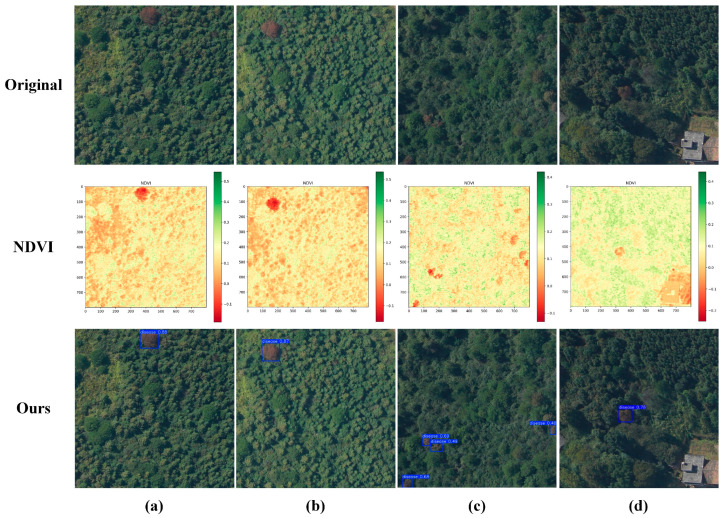
NDVI and detection result. (**a**–**d**) Four pairs of experimental results.

At the same time, to enhance the intuitiveness and interpretability of the model’s detection results, Figure 4 presents the original UAV remote sensing imagery, the normalized difference vegetation index (NDVI) image, and the target detection result image generated by the SLMW-Net model. These three types of images are precisely aligned spatially, clearly demonstrating the high spatial consistency between the suspected pine wilt disease infection areas identified by the model and the regions with low NDVI values. Specifically, the NDVI image reflects the physiological health status of vegetation within the study area, where regions with significantly low NDVI values typically indicate severe vegetation stress, degradation, or even non-vegetated areas. The bounding boxes predicted by SLMW-Net are highly overlapped with these low NDVI regions, indicating that the model exhibits strong biological relevance and spatial localization accuracy in identifying diseased areas.

### 3.4. Module Effectiveness Experiment

We propose three modules, namely SFEM, MFAM, and WLIoU Loss functions. To evaluate the performance of these modules, we first compare the SFEM module with other feature extraction modules. Subsequently, the MFAM module is evaluated and compared with various attention mechanisms. The WLIoU Loss function is compared with other commonly used loss functions. Under consistent experimental settings, the feasibility and performance of the proposed modules are verified by integrating each module into SLMW-Net for comparison.

#### 3.4.1. Effectiveness of SFEM

In this research, we introduce the SFEM module designed to dynamically extract disease features from UAV remote sensing images of pine forests. By integrating learnable normalization, the SFEM module improves the representation of regions affected by nematodes, effectively overcoming the challenge of feature extraction in complex background environments. For comparison of the SFEM module’s performance, we conducted comparison experiments on Fourier Convolution (FFC) [41], Pyramid Convolution (PyConv) [42], and Multi-scale Convolution (MSCA) [43]. The comparison results are presented in Table 2. The research results show that both the SFEM and Fourier Convolution modules substantially improve detection performance for pine wilt disease. Although Fourier Convolution is known for its simplicity, flexibility, and computational efficiency, it is useful in distinguishing targets from complex backgrounds. Challenges exist that may reduce detection accuracy in practical applications. Consequently, the SFEM module is integrated into the backbone to improve both performance and efficiency in detecting pine wilt disease in visually complex scenarios.

#### 3.4.2. Effectiveness of MFAM

In this research, we introduce a novel attentional mechanism, MFAM, aimed at improving the feature representation of pine wood nematode infestations captured in UAV images. By improving the ability to extract subtle infestation features, MFAM significantly boosts recognition accuracy. To validate its effectiveness, we compared MFAM with three existing attentional mechanisms: CBAM [44], Star Blocks [45], and SimAM [46], with the results presented in Table 3. Specifically, CBAM applies channel and spatial attention mechanisms separately to weight features. However, this approach may overlook the interdependencies between channel and spatial information, and the independent use of spatial and channel attention could result in incomplete feature representation. SimAM, while not increasing the number of network parameters, struggles to capture fine details and subtle features, particularly in the context of pine wilt disease detection. Star Blocks improves feature processing efficiency by mapping inputs to a high-dimensional nonlinear feature space via a star operation without expanding the network. Nevertheless, it does not perform as effectively as MFAM in extracting minor features of pine wilt disease. Our proposed MFAM module effectively captures fine details by integrating Grouped Attention with a Gated Feed-Forward network, thereby enhancing the ability to extract subtle features and improving its overall performance in detecting pine wood nematode infestation.

#### 3.4.3. Effectiveness of WLIoU Loss

In this study, the proposed loss function, WLIoU Loss, integrates moderating factors with linear stretching and truncation mechanisms, enabling an equilibrium between detection performance and model complexity. The impact of various IoU loss functions on nematode disease detection is shown in Table 4. It shows that using WLIoU Loss leads to improved performance metrics for the model, emphasizing its effectiveness in localizing nematode lesions. Additionally, we assessed the performance of CIoU [47] and GIoU [48]. The results indicate that WLIoU Loss outperforms both GIoU and CIoU, particularly in handling challenging samples, as it better captures the distribution characteristics of pine wilt disease.

### 3.5. Ablation Experiment

In this research, we introduce SFEM, MFAM, and WLIoU Loss into the SLMW-Net model. SFEM enhances understanding of both the representation of pine trees and their spatial characteristics through convolution and learnable normalization layers. This improvement enables the model to concentrate on key features of infected pine trees in complex backgrounds, thereby increasing the accuracy of detecting diseased pine trees in diverse natural environments. MFAM emphasizes subtle features of pine trees initially infected with nematode disease, thereby enhancing the ability to perceive infected trees and extract fine details. WLIoU Loss facilitates the update of the model’s parameters through loss computation and adjusts the weights of positive and negative samples, thereby improving generalization ability and overall performance. Table 5 shows the outcomes of the ablation experiment. The data demonstrate that SLMW-Net achieves the highest performance across all key metrics, with an mAP@0.5 of 86.7%, Recall of 80.6%, Precision of 80.5%, and mAP@0.5:0.95 reaching 40.1%. These results validate the substantial advantages of SFEM, MFAM, and WLIoU Loss. Specifically, the combined application of these modules in SLMW-Net produces a cumulative effect, enabling the model to better differentiate the target from its surroundings, especially in complex image scenarios. In scenes with intricate or similar features, SFEM, MFAM, and WLIoU Loss effectively reduce misidentifications, efficiently enhance attention to subtle initial features, and ensure highly accurate target detection.

### 3.6. Comparison with State-of-the-Art Methods

#### 3.6.1. SLMW-Net Performance on ARen Dataset

To thoroughly assess our model, we carry out comparison experiments with seven target detection approaches under identical testing conditions. These include Faster R-CNN [18], YOLOv3 [49], YOLOv5s [50], YOLOv7 [51], YOLOv8n [52], YOLOv9m [53], and YOLOv10n [54], allowing for a thorough analysis of the performance of SLMW-Net. Table 6 displays the results. In the two-stage detection model, candidate frames are initially generated using selective search. Features are then extracted from the input image using a pre-trained CNN, and target prediction is performed via a support vector machine. However, because each candidate frame requires separate feature extraction, this results in the generation of numerous redundant features, ultimately increasing detection time. Faster R-CNN addresses this issue by integrating candidate frame generation, classification, and regression into a single network, enabling joint training and significantly accelerating both the training and detection processes. Despite this, Faster R-CNN encounters difficulties in detecting small objects. Based on the results shown in Table 6, we observe that the one-stage detection models outperform the two-stage Faster R-CNN model in terms of overall performance. At the same time, Params and FLOPs serve as crucial metrics for a comprehensive evaluation of both the model size and the computational cost during inference. SLMW-Net shows advantages over other lightweight models in terms of the balance between computing overhead and performance. While lightweight models like YOLOv8n and YOLOv10n perform well in terms of computational efficiency, SLMW-Net makes a thorough trade-off between inspection accuracy and computing requirements. Overall, SLMW-Net optimizes both Params and FLOPs, effectively reducing computational complexity without sacrificing accuracy.

**Figure 5:**
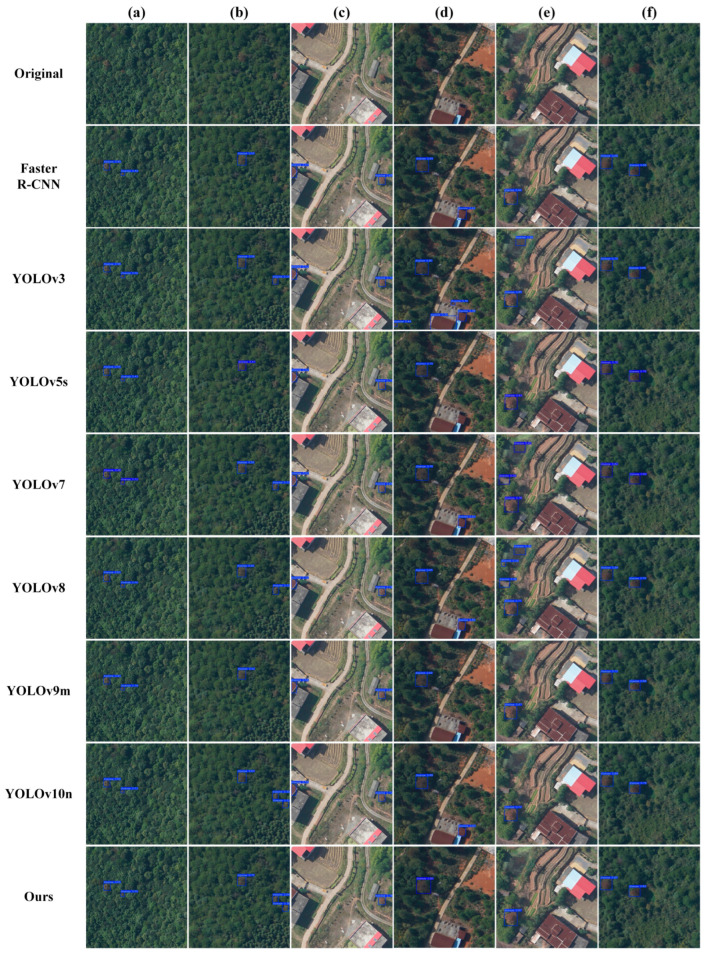
Comparison results of ARen dataset. (**a**–**f**) Six samples of ARen dataset.

The visualization of the experimental results is shown in Figure 5. Figure 5a,b display pine trees initially infected with pine wilt disease. It can be seen that our proposed SLMW-Net achieves optimal performance, successfully attaining the highest detection accuracy without any misdetections. Figure 5c–e demonstrate the detection in a complex background. In Figure 5c, the other seven models experienced misdetections, incorrectly identifying houses as pine trees infected with pine wilt disease, while SLMW-Net did not experience misdetections and attained the highest accuracy among the correctly detected targets. In Figure 5d, all models except YOLOv9m experienced misdetections. Although YOLOv9m did not misdetect any targets, its detection accuracy was lower than that of our proposed SLMW-Net. YOLOv3, although having accuracy consistent with SLMW-Net for correctly detected targets, still had four misdetections. In Figure 5e, some features resembling pine wilt disease were mistakenly identified as infected pine trees by YOLOv3, YOLOv7, and YOLOv8n. Although Faster R-CNN, YOLOv5s, YOLOv9m, and YOLOv10 did not experience misdetections, their detection accuracies were lower than that of SLMW-Net. Figure 5f shows a more obvious pine wood nematode infestation, where the detection accuracy of the other models was not as high as that of our proposed SLMW-Net. In summary, SLMW-Net is able to more effectively extract the complex features of pine wilt disease, achieve higher precision detection, and meet the required accuracy for pine wilt disease detection.

#### 3.6.2. SLMW-Net Performance on Roboflow Dataset

To further evaluate the performance of SLMW-Net, we obtain a public dataset for pine wilt disease detection from the Roboflow platform https://universe.roboflow.com/project-dkq3q/-9pmdt/dataset/8 (accessed on 16 June 2025). This dataset includes 4301 training images, 412 validation images, and 202 test images, each with a resolution of 640 × 640 pixels. The training images are adjusted for brightness in the range of −15% to +15%. We screen the images in this dataset and verify the performance with seven other models under the same experimental environment. Compared to the other models in Table 7, SLMW-Net performs the best in detecting pine wilt disease. We attribute the performance advantage of SLMW-Net to the following aspects: (a) SLMW-Net improves upon YOLOv11, incorporating several advanced technologies to enhance the model’s accuracy and speed while ensuring its robust detection performance; (b) SFEM, a key component of the feature extraction module, significantly enhances ability to extract complex features of pine wilt disease and improves its ability to distinguish features in non-forested areas; (c) MFAM, an attention mechanism introduced before the neck and the integration of GTM multiple times within the neck, successfully strikes a balance between global and local features, enabling the feature map to express its characteristics more clearly, especially the subtle changes in pine wilt disease; (d) WLIoU Loss optimizes the training performance by effectively adjusting the weights of different samples, balancing fitting ability and generalization capacity.

## 4. Discussion

**Figure 6:**
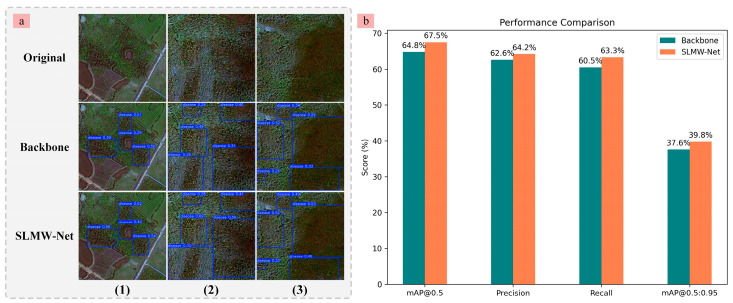
Performance of backbone and SLMW-Net on satellite images. (**a**) Visualization results; (**b**) Data statistics.

The proposed SLMW-Net model in this study is primarily designed for pine wilt disease detection using UAV remote sensing imagery. To assess the model’s potential for satellite remote sensing applications, we carried out experiments utilizing Gaofen-2 satellite imagery, incorporating the blue, green, and red bands, to examine its generalization ability and real-world performance across various data sources. Specifically, we obtained 6424 images for training, 803 images for testing, and 803 images for validation, conducting a comparative analysis between the backbone and SLMW-Net models. Figure 6a shows that SLMW-Net retains a certain level of effectiveness in detecting regional PWD symptoms when applied to satellite imagery. Furthermore, Figure 6b indicates that, although the overall detection accuracy of SLMW-Net slightly declines on satellite images, primarily due to their relatively lower spatial resolution, it still demonstrates clear performance advantages over the backbone. The result not only validates the effectiveness of key architectural components within SLMW-Net on satellite remote sensing imagery but also further demonstrates the potential feasibility of this model in detecting regional pine wilt disease under satellite image conditions.

## 5. Conclusions

In order to explore the best method for detecting pine wilt disease, we propose an SLMW-Net accurate pine wilt disease detection method based on UAV images. First, we propose an SFEM module to enhance the feature extraction capability for detecting pine wilt disease in complex backgrounds. Then, the feature information is enhanced using MFAM to direct the model to focus on tiny features. Finally, WLIoU Loss was designed to calculate the loss of the model and guide the model parameter update. As a result, the method overcomes the problems of leakage and misdetection in pine wilt disease detection and improves the detection accuracy. In addition, SLMW-Net better matches the data features of UAV pine forest images. By enabling the timely and accurate detection of pine wilt disease, this study can assist producers in developing targeted control measures based on the identified nematode infection. In the experimental part, we divided 750 images collected in Anren, Hunan Province, into an 8:2 training set and validation set. As evidenced by the experimental results, SLMW-Net greatly improves the detection accuracy while maintaining the standard detection speed, and it outperforms other detection models. Meanwhile, we tested it on the public dataset of the Roboflow platform and found that our model still outperforms all other models, thus proving the effectiveness of our proposed model.

Despite promising results, several issues remain. Our primary dataset is geographically restricted to a single county, and while external validation with public datasets has been conducted, further validation is needed in regions with diverse climatic and ecological conditions. Additionally, early stage disease symptoms are often not visible, making it difficult to detect infections from images alone. To address these issues, future studies will concentrate on broadening the dataset to encompass multiple regions and incorporating IoT sensor technology with deep learning models to improve disease detection and prediction. The effectiveness of the model will also be assessed across different time periods and epidemic cycles to gain deeper insights into how temporal factors affect its accuracy and generalizability. Overcoming these limitations will contribute to the development of a more reliable early warning detection system, capable of effectively supporting forest management and ecological conservation in various environments.

## Figures and Tables

**Table 1:** Hardware and software environment settings.

Category	Component	Specification
Hardware	CPU	AMD EPYC 9754 128-Core Processor
	RAM	1TB
	GPU	NVIDIA GeForce RTX 4090 D
Software	OS	Linux
	Python	Python 3.8.10
	CUDA Toolkit	11.8
	CUDNN	V8.7
	PyTorch	2.0.0

**Table 2:** The experimental findings regarding the effectiveness of SFEM.

Methods	mAP@0.5 (%)	Precision (%)	Recall (%)	mAP@0.5:0.95 (%)
FFC	84.2	77.3	77.7	38.5
PyConv	83.9	76.8	75.6	38.2
MSCA	82.6	75.5	73.0	37.6
SFEM	84.7	77.8	78.9	38.8

**Table 3:** The experimental findings regarding the effectiveness of MFAM.

Methods	mAP@0.5 (%)	Precision (%)	Recall (%)	mAP@0.5:0.95 (%)
CBAM	83.1	75.9	74.8	37.2
SimAM	83.6	77.8	76.2	37.8
Star Blocks	84.2	76.4	75.3	38.3
MFAM	84.9	78.9	76.3	38.9

**Table 4:** The experimental findings regarding the effectiveness of WLIoU Loss.

Methods	mAP@0.5 (%)	Precision (%)	Recall (%)	mAP@0.5:0.95 (%)
CIOU	83.6	77.3	78.7	37.6
GIOU	83.2	76.2	77.1	37.4
WLIoU Loss	84.6	78.6	78.9	38.8

**Table 5:** Ablation experiment.

A	B	C	mAP@0.5 (%)	Precision (%)	Recall (%)	mAP@0.5:0.95 (%)
no	no	no	83.9	76.9	76.3	38.4
yes	no	no	84.7	77.8	78.9	38.8
no	yes	no	84.9	78.9	76.3	38.9
no	no	yes	84.6	78.6	78.9	38.8
yes	yes	no	85.2	78.7	78.2	39.6
yes	no	yes	85.4	79.2	79.4	39.5
no	yes	yes	85.5	80.4	78.8	39.1
yes	yes	yes	86.7	80.5	80.6	40.1

**Table 6:** Compare with other models on ARen dataset.

Methods	mAP@0.5 (%)	Precision (%)	Recall (%)	mAP@0.5:0.95 (%)	Params (M)	FLOPs (G)
Faster R-CNN	59.5	58.5	61.3	23.2	28.3	492.9
YOLOv3	82.2	75.7	75.7	39.3	61.5	155.3
YOLOv5s	83.4	76.4	77.7	37.3	7.0	15.9
YOLOv7	79.7	74.8	74.9	37.4	37.2	105.1
YOLOv8n	83.7	78.0	74.0	38.3	3.0	8.2
YOLOv9m	83.4	79.5	74.6	39.9	20.2	77.5
YOLOv10n	80.9	78.9	71.8	37.7	2.7	8.4
SLMW-Net	86.7	80.5	80.6	40.1	3.9	8.8

**Table 7:** Compare with other models on Roboflow dataset.

Methods	mAP@0.5 (%)	Precision (%)	Recall (%)	mAP@0.5:0.95 (%)	Params (M)	FLOPs (G)
Faster R-CNN	71.0	54.4	73.8	31.2	28.3	492.9
YOLOv3	81.6	78.0	75.9	37.4	61.5	155.3
YOLOv5s	83.7	79.4	75.5	38.4	7.0	15.9
YOLOv7	84.6	78.9	79.8	38.0	37.2	105.1
YOLOv8n	85.1	80.6	78.2	39.3	3.0	8.2
YOLOv9m	84.0	77.7	77.1	39.4	20.2	77.5
YOLOv10n	82.3	77.4	75.2	38.2	2.7	8.4
SLMW-Net	85.3	81.3	80.7	40.4	3.9	8.8

## Data Availability

The data related to the findings of this research are available upon request from the corresponding author.

## References

[1] Pan S., Yan P., Zhao R., Li F., Wang L., Wang Y., Lv Z., Ma Y., Yu M., Guo X. (2025). Conduction of AgNPs with different surface charges in pine trees and their prevention and control of pine wood nematode disease. Chem. Eng. J..

[2] Luo J., Fan J., Huang S., Wu S., Zhang F., Li X. (2025). Semi-supervised learning techniques for detection of dead pine trees with UAV imagery for pine wilt disease control. Int. J. Remote Sens..

[3] Khuman S.N., Lee H.Y., Cho I.-G., Chung D., Lee S.Y., Lee J., Oh J.-K., Choi S.-D. (2024). Monitoring of Organochlorine Pesticides Using Pine Needle, Pine Bark, and Soil Samples across South Korea: Source Apportionment and Implications for Atmospheric Transport. Chemosphere.

[4] Lin H., Ning L., Dai T., Wei W., Ma N., Hong M., Wang F., You C. (2024). Application of Bio-Based Nanopesticides for Pine Surface Retention and Penetration, Enabling Effective Control of Pine Wood Nematodes. Chem. Eng. J..

[5] Gao R., Liu L., Li R., Fan S., Dong J., Zhao L. (2023). Predicting Potential Distributions of Monochamus Saltuarius, a Novel Insect Vector of Pine Wilt Disease in China. Front. For. Glob. Change.

[6] Hao Z., Huang J., Li X., Sun H., Fang G. (2021). A Multi-Point Aggregation Trend of the Outbreak of Pine Wilt Disease in China over the Past 20 Years. For. Ecol. Manag..

[7] Back M.A., Bonifácio L., Inácio M.L., Mota M., Boa E. (2024). Pine Wilt Disease: A Global Threat to Forestry. Plant Pathol..

[8] Li M., Li H., Ding X., Wang L., Wang X., Chen F. (2022). The Detection of Pine Wilt Disease: A Literature Review. Int. J. Mol. Sci..

[9] Wang W., Zhu Q., He G., Liu X., Peng W., Cai Y. (2023). Impacts of Climate Change on Pine Wilt Disease Outbreaks and Associated Carbon Stock Losses. Agric. For. Meteorol..

[10] Sharma A., Cory B., McKeithen J., Frazier J. (2020). Structural Diversity of the Longleaf Pine Ecosystem. For. Ecol. Manag..

[11] You J., Zhang R., Lee J. (2021). A Deep Learning-Based Generalized System for Detecting Pine Wilt Disease Using RGB-Based UAV Images. Remote Sens..

[12] Syifa M., Park S.-J., Lee C.-W. (2020). Detection of the Pine Wilt Disease Tree Candidates for Drone Remote Sensing Using Artificial Intelligence Techniques. Engineering.

[13] Pan J., Lin J., Xie T. (2023). Exploring the Potential of UAV-Based Hyperspectral Imagery on Pine Wilt Disease Detection: Influence of Spatio-Temporal Scales. Remote Sens..

[14] Iordache M.-D., Mantas V., Baltazar E., Pauly K., Lewyckyj N. (2020). A Machine Learning Approach to Detecting Pine Wilt Disease Using Airborne Spectral Imagery. Remote Sens..

[15] Yu R., Luo Y., Zhou Q., Zhang X., Wu D., Ren L. (2021). A Machine Learning Algorithm to Detect Pine Wilt Disease Using UAV-Based Hyperspectral Imagery and LiDAR Data at the Tree Level. Int. J. Appl. Earth Obs. Geoinf..

[16] Hu G., Wang T., Wan M., Bao W., Zeng W. (2022). UAV Remote Sensing Monitoring of Pine Forest Diseases Based on Improved Mask R-CNN. Int. J. Remote Sens..

[17] Li F., Liu Z., Shen W., Wang Y., Wang Y., Ge C., Sun F., Lan P. (2021). A Remote Sensing and Airborne Edge-Computing Based Detection System for Pine Wilt Disease. IEEE Access.

[18] Ren S., He K., Girshick R., Sun J. (2017). Faster R-CNN: Towards Real-Time Object Detection with Region Proposal Networks. IEEE Trans. Pattern Anal. Mach. Intell..

[19] Liu W., Anguelov D., Erhan D., Szegedy C., Reed S.E., Fu C.-Y., Berg A.C. (2016). SSD: Single Shot MultiBox Detector. Computer Vision—ECCV 2016.

[20] Redmon J., Divvala S., Girshick R., Farhadi A. You Only Look Once: Unified, Real-Time Object Detection. Proceedings of the 2016 IEEE Conference on Computer Vision and Pattern Recognition.

[21] Redmon J., Farhadi A. YOLO9000: Better, Faster, Stronger. Proceedings of the 2017 IEEE Conference on Computer Vision and Pattern Recognition (CVPR).

[22] Wu K., Zhang J., Yin X., Wen S., Lan Y. (2023). An Improved YOLO Model for Detecting Trees Suffering from Pine Wilt Disease at Different Stages of Infection. Remote Sens. Lett..

[23] Bochkovskiy A., Wang C.-Y., Liao H.-Y.M. (2020). YOLOv4: Optimal speed and accuracy of object detection. arXiv.

[24] Sun Z., Ibrayim M., Hamdulla A. (2022). Detection of Pine Wilt Nematode from Drone Images Using UAV. Sensors.

[25] Wang L., Cai J., Wang T., Zhao J., Gadekallu T.R., Fang K. (2024). Detection of Pine Wilt Disease Using AAV Remote Sensing with an Improved YOLO Model. IEEE J. Sel. Top. Appl. Earth Obs. Remote Sens..

[26] Ye X., Pan J., Shao F., Liu G., Lin J., Xu D., Liu J. (2024). Exploring the Potential of Visual Tracking and Counting for Trees Infected with Pine Wilt Disease Based on Improved YOLOv5 and StrongSORT Algorithm. Comput. Electron. Agric..

[27] Zhu X., Wang R., Shi W., Liu X., Ren Y., Xu S., Wang X. (2024). Detection of Pine-Wilt-Disease-Affected Trees Based on Improved YOLO V7. Forests.

[28] Huang X., Gang W., Li J., Wang Z., Wang Q., Liang Y. (2024). Extraction of Pine Wilt Disease Based on a Two-Stage Unmanned Aerial Vehicle Deep Learning Method. J. Appl. Remote Sens..

[29] Yuan J., Wang L., Wang T., Bashir A.K., Al Dabel M.M., Wang J., Feng H., Fang K., Wang W. (2025). YOLOv8-RD: High-Robust Pine Wilt Disease Detection Method Based on Residual Fuzzy YOLOv8. IEEE J. Sel. Top. Appl. Earth Obs. Remote Sens..

[30] Xu S., Huang W., Wang D., Zhang B., Sun H., Yan J., Ding J., Wang J., Yang Q., Huang T. (2024). Automatic Pine Wilt Disease Detection Based on Improved YOLOv8 UAV Multispectral Imagery. Ecol. Inform..

[31] Yao J., Song B., Chen X., Zhang M., Dong X., Liu H., Liu F., Zhang L., Lu Y.-B., Xu C. (2024). Pine-YOLO: A Method for Detecting Pine Wilt Disease in Unmanned Aerial Vehicle Remote Sensing Images. Forests.

[32] Khanam R., Hussain M. (2024). YOLOv11: An Overview of the Key Architectural Enhancements. arXiv.

[33] Deng Y., Xi H., Zhou G., Chen A., Wang Y., Li L., Hu Y. (2023). An Effective Image-Based Tomato Leaf Disease Segmentation Method Using MC-UNet. Plant Phenomics.

[34] Wan L., Zhu W., Dai Y., Zhou G., Chen G., Jiang Y., Zhu M., He M. (2024). Identification of Pepper Leaf Diseases Based on TPSAO-AMWNet. Plants.

[35] Li J., Zhao F., Zhao H., Zhou G., Xu J., Gao M., Li X., Dai W., Zhou H., Hu Y. (2024). A Multi-Modal Open Object Detection Model for Tomato Leaf Diseases with Strong Generalization Performance Using PDC-VLD. Plant Phenomics.

[36] Hang R., Li Z., Liu Q., Ghamisi P., Bhattacharyya S.S. (2021). Hyperspectral Image Classification with Attention-Aided CNNs. IEEE Trans. Geosci. Remote Sens..

[37] Sadasivan A., Gananathan K., Dhanith J., Balasubramanian S. (2025). A Systematic Survey of Graph Convolutional Networks for Artificial Intelligence Applications. Wiley Interdiscip. Rev. Data Min. Knowl. Discov..

[38] Hu Y., Zhang Y., Liu S., Zhou G., Li M., Hu Y., Li J., Sun L. (2024). DMFGAN: A Multifeature Data Augmentation Method for Grape Leaf Disease Identification. Plant J..

[39] Liu Z., Zhou G., Zhu W., Chai Y., Li L., Wang Y., Hu Y., Dai W., Liu R., Sun L. (2024). Identification of Rice Disease under Complex Background Based on PSOC-DRCNet. Expert Syst. Appl..

[40] Putra H.A.A., Murni Arymurthy A., Chahyati D. (2025). Enhancing Bounding Box Regression for Object Detection: Dimensional Angle Precision IoU-Loss. IEEE Access.

[41] Chu T., Chen J., Sun J., Lian S., Wang Z., Zuo Z., Zhao L., Xing W., Lu D. Rethinking Fast Fourier Convolution in Image Inpainting. Proceedings of the 2023 IEEE/CVF International Conference on Computer Vision (ICCV).

[42] Duta I.C., Liu L., Zhu F., Shao L. (2020). Pyramidal Convolution: Rethinking Convolutional Neural Networks for Visual Recognition. arXiv.

[43] Guo M.-H., Lu C.-Z., Hou Q., Liu Z., Cheng M.-M., Hu S.-M. (2022). SegNeXt: Rethinking Convolutional Attention Design for Semantic Segmentation. Adv. Neural Inf. Process. Syst..

[44] Woo S., Park J., Lee J.-Y., Kweon I.S., Ferrari V., Hebert M., Sminchisescu C., Weiss Y. (2018). CBAM: Convolutional Block Attention Module. Computer Vision—ECCV 2018.

[45] Ma X., Dai X., Bai Y., Wang Y., Fu Y. Rewrite the Stars. Proceedings of the 2024 IEEE/CVF Conference on Computer Vision and Pattern Recognition (CVPR).

[46] Yang L., Zhang R.-Y., Li L., Xie X. SimAM: A Simple, Parameter-Free Attention Module for Convolutional Neural Networks. Proceedings of the 38th International Conference on Machine Learning.

[47] Zheng Z., Wang P., Ren D., Liu W., Ye R., Hu Q., Zuo W. (2022). Enhancing Geometric Factors in Model Learning and Inference for Object Detection and Instance Segmentation. IEEE Trans. Cybern..

[48] Rezatofighi H., Tsoi N., Gwak J., Sadeghian A., Reid I., Savarese S. Generalized Intersection Over Union: A Metric and a Loss for Bounding Box Regression. Proceedings of the IEEE/CVF Conference on Computer Vision and Pattern Recognition (CVPR).

[49] Redmon J., Farhadi A. (2018). YOLOv3: An Incremental Improvement. arXiv.

[50] Khanam R., Hussain M. (2024). What Is YOLOv5: A Deep Look into the Internal Features of the Popular Object Detector. arXiv.

[51] Wang C.-Y., Bochkovskiy A., Liao H.-Y.M. YOLOv7: Trainable Bag-of-Freebies Sets New State-of-the-Art for Real-Time Object Detectors. Proceedings of the 2023 IEEE/CVF Conference on Computer Vision and Pattern Recognition (CVPR).

[52] Varghese R., M. S. YOLOv8: A Novel Object Detection Algorithm with Enhanced Performance and Robustness. Proceedings of the 2024 International Conference on Advances in Data Engineering and Intelligent Computing Systems (ADICS).

[53] Wang C.-Y., Yeh I.-H., Liao H.-Y.M. (2024). YOLOv9: Learning What You Want to Learn Using Programmable Gradient Information. arXiv.

[54] Wang A., Chen H., Liu L., Chen K., Lin Z., Han J., Ding G. (2024). YOLOv10: Real-Time End-to-End Object Detection. arXiv.

